# A Case

**Published:** 1863-09

**Authors:** 


### A CASE.

ABOUT fifteen months ago, in excavating a lower bicuspid, the pulp was exposed and wounded. The bleeding was arrested by a solution of tannin, and the tooth was filled with "Hill's Stopping." To-day the filling was removed, the pulp was alive and healthy, still freely exposed, and highly sensitive when touched with a smooth probe. A thin cap of "Hill's Stopping" was laid over the pulp and the tooth was filled with gold. W.

